# Looking for Biomarkers Which May Explain Idiopathic Environmental Intolerance Attributed to Electromagnetic Fields (IEI‐EMF): Does RF‐EMF Exposure Influence Salivary Cortisol Response?

**DOI:** 10.1002/bem.70021

**Published:** 2025-09-05

**Authors:** Adam Verrender, Jacob Manley, Nikkeah K. Wallace, Sarah P. Loughran, Rodney J. Croft

**Affiliations:** ^1^ Australian Centre for Electromagnetic Bioeffects Research Wollongong Australia; ^2^ School of Psychology, Faculty of Arts, Social Sciences, and Humanities University of Wollongong Wollongong Australia; ^3^ Australian Radiation Protection and Nuclear Safety Agency Yallambie Australia

**Keywords:** electromagnetic fields, electromagnetic hypersensitivity, non‐specific symptoms, radiofrequency

## Abstract

In order to understand Idiopathic Environmental Intolerance attributed to Electromagnetic Fields (IEI‐EMF), it has been argued that it is crucial to test for effects of radiofrequency electromagnetic fields (RF‐EMF) on biomarkers, given that they can be more objective than symptom reports. While no clear evidence links RF‐EMF exposure to biomarker changes, research remains limited and largely speculative due to the lack of known bioeffect mechanisms. However, there is in vitro evidence that cortisol is affected by heating, which, as RF‐EMF causes heating, raises the possibility that RF‐EMF exposure may increase cortisol via thermally mediated processes. If cortisol is affected by RF‐EMF exposure, it may form part of a broader aetiology of IEI‐EMF, where RF‐EMF‐induced physiological (cortisol) inputs first generate somatic sensations, which are then fostered by expectancy or learning‐based processes to generate symptoms. However, studies investigating whether RF‐EMF exposure influences cortisol have reported inconsistent, but mostly null results, and many suffer from methodological issues. The current study was designed with several methodological improvements to determine whether RF‐EMF affects cortisol. Seventy‐two participants completed a randomized, double‐blind, counterbalanced provocation study where they were exposed to both active (2 W/kg peak SAR_10g_ in head) and sham RF‐EMF (0 W/kg peak SAR_10g_ in head). Despite implementing several methodological improvements, the current study failed to find an effect of RF‐EMF exposure on salivary cortisol concentration. This study provides a valuable direction for future research and stresses the importance of establishing and testing theoretically plausible interactions between low‐level RF‐EMF exposure, the human body, and IEI‐EMF symptoms.

## Summary

- The current study incorporated several methodological improvements to test whether exposure to RF‐EMF increases salivary cortisol concentration in humans.
- The current study failed to find an effect of RF‐EMF exposure on salivary cortisol concentration, and there was no influence of participants' sex.
- Given the novelty of the methodological improvements implemented in this study, this lack of effect on salivary cortisol is very robust.

## 1 Introduction

Idiopathic Environmental Intolerance attributed to Electromagnetic Fields (IEI‐EMF), also known as Electromagnetic Hypersensitivity (EHS), is a controversial condition where people report experiencing a broad range of non‐specific symptoms which they claim are caused by exposure to the radiofrequency electromagnetic fields (RF‐EMF) emitted by everyday electronic and wireless technologies. Like other IEI's, there is no consistent pattern of either the types of symptoms reported, their duration, their severity, or the types of exposure emitting devices purported to cause the symptoms (Baliatsas et al. 2012). To represent what is commonly observed amongst individuals reporting IEI conditions, Haanes et al. (2020) has proposed using the broader term Symptoms Associated with Environmental Factors (SAEF). Although some previous studies have estimated the prevalence of IEI‐EMF at approximately 5% (Schreier et al. 2006; Huang et al. 2018), the rapidly changing nature of exposure patterns and sources, the heterogeneity of the condition, inconsistencies in the methods used by epidemiological studies, and the scarcity of recent data make it difficult to provide either an accurate or up‐to‐date estimate of the prevalence of the condition.

While there is no doubt that the symptoms experienced by those who report IEI‐EMF are disabling and can substantially impact daily functioning (Pitron et al. 2023), to date, the scientific evidence has failed to find any relationship between exposure to RF‐EMF and reported symptoms (de Vocht and Röösli 2025). For instance, well‐controlled double‐blind provocation studies, where participants are exposed to both active and sham RF‐EMF, have consistently shown that sham exposures and/or a belief of being exposed are sufficient to trigger symptoms (Hillert et al. 2008; Ledent et al. 2025; Oftedal et al. 2007; Rubin et al. 2010; Verrender et al. 2018a). This indicates that the condition is more closely related to a nocebo response, where conscious or subconscious expectations trigger or exacerbate symptoms (Bosch‐Capblanch et al. 2024; Rubin et al. 2010).

Despite this, some researchers and many IEI‐EMF advocacy groups strongly reject the notion that IEI‐EMF is psychogenic in origin, and instead argue that the condition is *caused* by RF‐EMF exposure, citing evidence of differences in a range of biomarkers in those reporting IEI‐EMF (e.g., Belpomme et al. 2015; Irigaray et al. 2018). However, in addition to suffering from important methodological issues (e.g., failure to control for multiple comparisons and thus chance findings), those studies did not test for effects of RF‐EMF. Instead, they merely tried to identify whether there were biomarker abnormalities in people who report experiencing IEI‐EMF, regardless of whether the markers were affected by RF‐EMF. Accordingly, these studies cannot be used to support the hypothesis that IEI‐EMF is caused by RF‐EMF exposure. It is understandable that such exploratory approaches have been undertaken in place of experimental research given the lack of relevant RF‐EMF biophysical mechanisms with which to guide the endeavor. However, it is known that RF‐EMF heats tissue, and although the general scientific consensus has been that this heating (< 0.1°C) by RF‐EMF exposure within international exposure guidelines is too small to induce cell damage or elicit a physiological response (Repacholi 1998), we recently found RF‐EMF exposure caused thermoregulatory change, as evidenced via vasodilation of the fingers (Loughran et al. 2019). This indicates that exposure to RF‐EMF within the current exposure guidelines is sufficient to engage thermoregulatory processes, raising the possibility that heating is a sufficient biophysical mechanism to engage physiological processes that may impact on subjective symptoms and thus IEI‐EMF. Importantly, as argued by Van den Bergh et al. (2017), a physiological response of this nature does not in itself need to be sufficient to trigger IEI‐EMF symptoms. Instead, it may form part of a broader process involving both cognitions and physiology, whereby a combination of cognitive and physiological inputs first generates somatic sensations, which are then utilized by expectancy and/or conditioning‐based processes in the development of symptoms (Van den Bergh et al. 2021).

Interestingly, increases in temperature in vitro have been shown to affect the concentration of bioavailable free cortisol (Cameron et al. 2010; Lewis et al. 2005). Cortisol is commonly known as the stress hormone for its role in regulating arousal and the stress response. However, it is also involved in metabolism, immune response, and cognitive and affective functioning (Kumsta et al. 2007). Approximately 80%–90% of the body's cortisol is attached to corticosteroid‐binding globulin (CBG), leaving only a small amount available to bind to cortisol receptor sites (Kumsta et al. 2007; Westphal 1983). However, CBG's affinity for cortisol is prone to fluctuations in temperature. For instance, Cameron et al. (2010) found that cortisol's affinity for CBG was heavily influenced by temperature in vitro, where, as temperature increased from 37°C to 41°C, free cortisol concentration increased by 250%. This raises the possibility that RF‐EMF‐induced changes in body temperature could influence free‐cortisol concentration.

However, whether RF‐EMF‐derived heating can affect cortisol in humans remains unclear, as studies assessing cortisol response during and following exposure to RF‐EMF have yielded contradictory, but mostly null results, and many suffer from methodological limitations (Selmaoui and Touitou 2021). For instance, although Mann et al. (1998) reported a small, transient elevation in cortisol production during an overnight RF‐EMF exposure compared to sham, there was no overall effect of RF‐EMF exposure on cortisol, and the overnight effect was not replicated in a subsequent study (Radon et al. 2001). Similarly, Augner et al. (2010) reported an increase in salivary cortisol from a transition from a low to high RF‐EMF exposure emitted by a mobile phone base station. However, the results were not consistent across the study, and while the study claimed to increase ecological validity by being conducted in a field laboratory using a continually emitting base station, different exposure levels were produced by varying shielding provided via curtains, and no sham or no exposure condition was included. Conversely, Djeridane et al. (2008) reported that exposure to RF‐EMF for 2 h per day over a 4‐week period decreased cortisol concentration compared to baseline, however, that study was not double‐blinded, and it lacked a sham exposure condition. In a study comparing IEI‐EMF participants and healthy controls, Andrianome et al. (2017) did not find any difference in salivary cortisol concentration between the two groups. Although this may suggest the HPA axis is not affected in IEI‐EMF participants, the study did not utilize a provocation trial design, so is unable to determine if any potential differences between the two groups are a result of RF‐EMF exposure. While no other studies have found any effect of exposure to RF‐EMF on cortisol (Andrianome et al. 2019; Braune et al. 2002; De Seze et al. 2001; Ghosn et al. 2015; Radon et al. 2001), many of these also suffer from methodological limitations, including not controlling for diurnal variation in cortisol (Andrianome et al. 2019), using short exposure intervals (Andrianome et al. 2019), using single‐blind conditions (Braune et al. 2002), or testing small samples (Andrianome et al. 2019; Radon et al. 2001). Sex‐related differences have also been consistently demonstrated to influence cortisol response (Kudielka et al. 2009), however, these have not been accounted for in previous studies. As these limitations may have reduced the ability of previous studies to detect any effects, it remains unclear whether exposure to RF‐EMF influences salivary cortisol concentration.

To overcome these issues, the present study was designed with several methodological improvements to test the hypotheses that (1) active RF‐EMF exposure increases salivary cortisol response, and (2) any effect of RF‐EMF exposure on salivary cortisol response is related to sex differences. In particular, the study attempted to raise the chance of detecting larger effects by collecting salivary cortisol samples between 21 and 40 min of stimulus onset and conducting trials in the afternoon (to control for diurnal fluctuations in cortisol concentration) in line with the recommendations of Dickerson and Kemeny (2004). In addition to the randomized, double‐blind, counterbalanced protocol with a 15‐min exposure interval, whole saliva samples were collected via a passive drool method, which avoids any potential stress response induced by taking blood samples intravenously and allows for a consistent specimen as it avoids localized secretions of specific salivary glands. These improvements were implemented to increase sensitivity, and thus, the possibility of detecting potential effects. Given the lack of knowledge of the possible effect of RF‐EMF‐derived heating on cortisol response in humans, the present study has been designed to test for the presence of a small to moderate effect.

## 2 Materials and Methods

### 2.1 Participants

Seventy‐two participants (48 female, 24 male) aged 18–40 (*M* = 20.80, SD = 3.88) [in the following age categories: 18–29 (*n* = 69), 30–40 (*n* = 3)] were recruited through online advertisements, advertisements placed around the University of Wollongong campus, the University of Wollongong School of Psychology Research Participation Scheme, and word of mouth. An a priori power calculation conducted in G*Power 3.1 (Faul et al. 2007) determined that a minimum sample of 72 was required to detect a small‐moderate effect (*f* = 0.15). As there is a lack of evidence regarding the effect of heating on CBG's affinity for cortisol in intact organisms such as humans (as this has only been studied in vitro) and given the lack of understanding about RF‐EMF derived heat at a cellular level, it is impossible to accurately predict the size of an effect of RF‐EMF exposure on salivary cortisol based on previous literature. Therefore, the current study provides the power (0.80) to detect a main effect of RF‐EMF exposure and an interaction of RF‐EMF exposure and sex on cortisol concentration of greater than *f* = 0.15.

To be eligible for the study, participants were required to be between 18 and 55 years of age, be sufficiently fluent in English, and report being in good health. Participants who participated in previous studies in our laboratory were excluded from this study to minimize potential confounding. Participants were also excluded from the study if they reported that they were receiving treatment for or suffered from any acute or chronic illnesses, or if they reported taking any pharmaceutical (except the contraceptive pill) or illicit substances. Participants who were deemed suitable subsequently attended the University of Wollongong campus for one mutually convenient testing session. Informed, written consent was acquired from all participants. The study was approved by the University of Wollongong Human Research Ethics Committee (HE: 2023/081). Participants were compensated with a monetary gift card (general participants) or credit points (Psychology students). Data was collected between May 2023 and May 2024.

### 2.2 Design

**Figure 1:**
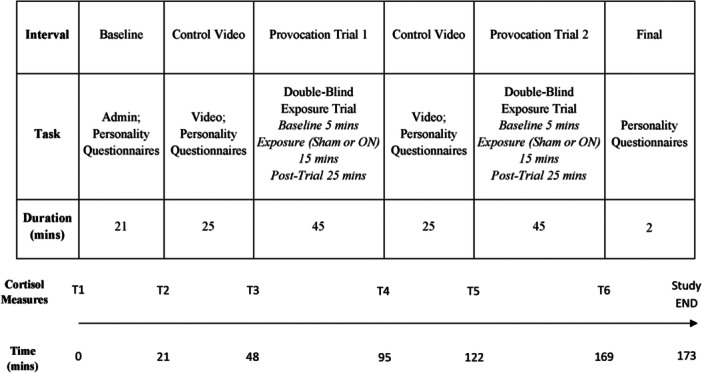
Timeline of the trial procedure outlining the six intervals (1st row), the tasks completed in each interval (2nd row), the duration of each interval (3rd row), the time point at which cortisol measures were taken (4th row), and the accumulated trial time (5th row).

A double‐blind, randomized, counter‐balanced, repeated measures design was utilized in this study. Each trial consisted of 6 intervals, including the Baseline, Control Video, Provocation Trial 1, Control Video, Provocation Trial 2, and Final interval, as outlined in Figure 1. Before attendance, for males and females separately, participants were pseudo‐randomly allocated into one of two experimental orders; “Sham then RF‐ON” (Group 1), or “RF‐ON then Sham” (Group 2), with the constraint that each sex had to have equal numbers of the two orders, and the same order could not be repeated sequentially (within sex) more than three times. To maintain double blinding, this process was completed by a researcher not involved in data collection (RC). To align with our previous research designs (Verrender et al. 2018; Verrender et al. 2025), all participants viewed two 20‐min segments of a science Question and Answer documentary titled “In Class with Brian Cox” (The Royal Institute of Australia 2014). This video contained an interview with an astrophysicist answering students' questions about the universe and contained no RF‐EMF related health content. The video was not relevant to any aspect of the hypothesis testing.

### 2.3 Radiofrequency Exposure

A 920 MHz GSM‐like signal (as emitted by a mobile phone in an active mode while transmitting voice) was generated using an sXh920 planar exposure system (IT'IS Foundation, Zurich, Switzerland). Two RF antennas placed on wooden pillars were positioned 42 mm vertically above the ear canal at a distance of 115 mm from the head (Loughran et al. 2019; Verrender et al. 2016). The RF exposure has been fully characterized and was calibrated to provide a peak‐spatial SAR averaged over 10 g of 0 W/kg and 2 W/kg for the Sham and RF‐ON conditions, respectively, [for full dosimetric data see Murbach et al. (2012)]. The sXh920 system enables precise exposure levels and has a failsafe mechanism to ensure that it cannot exceed the relevant RF general public exposure limits (ARPANSA RPS‐S1). The exposure was provided within a Faraday cage with approximately 80 dB attenuation at 920 MHz, which minimized any environmental EMF exposure from nearby RF EMF‐emitting devices such as mobile phones, Wi‐Fi, and mobile phone base stations.

### 2.4 Salivary Cortisol Measures

Whole saliva samples were collected using the passive drool method using SalivaBio 2 mL cryovials and Saliva Collection Aid (Salimetrics, USA). To capture peak cortisol change following the onset of a stimulus saliva samples were collected at six time points at least 21 min after the onset of each interval (except baseline) (Dickerson & Kemeny 2004), see Figure 1. For each provocation trial, the Baseline saliva sample was defined as the sample taken immediately following Control Video interval (T3 and T5), following a 25‐min period in which participants were passively viewing a video and completing personality questionnaires. Given there is no evidence of the amount of time it may take for RF‐EMF exposure to induce a cortisol change, to capture the potential peak cortisol change that may have occurred during the exposure, the Exposure saliva sample was defined as the sample taken 25 min after exposure ceased (T4 and T6).

Saliva samples were immediately stored in a freezer at −20°C and then transferred to a −80°C freezer immediately after cessation of the testing session. Whole saliva collected by passive drool is the gold standard when collecting oral fluid for biological testing. It avoids localized secretions of specific salivary glands (thus providing a more consistent specimen) and eliminates the risk of contamination or irritation associated with absorbent materials used in other collection methods, which may cause bleeding or uneven salivary gland activation. The samples were sent to Stratech Scientific salivary laboratory (Stratech Scientific APAC Pty Ltd, Sydney, Australia) for assay. On the day of assay, the samples were thawed to room temperature, centrifuged at 1500*g* for 15 min to collect clear saliva, and were analysed in duplicate using a commercially available ELISA assay (Salimetrics, USA) according to the manufacturer's instructions. The primary dependent variable of cortisol response in each provocation trial was calculated as a difference score (in μg/dL) between the baseline and exposure intervals; a difference score was used to minimize the influence of baseline variability.

### 2.5 Questionnaires

#### 2.5.1 Symptoms and Exposure Status Scale (SESS)

During the provocation trials, participants completed the SESS. Each item on the SESS was rated via pen and paper 100 mm visual analogue scales. To assess whether participants believed that exposure was on, the SESS asked participants “how sure are you of the current exposure status right now?” anchored with the terms “Definitely OFF” and “Definitely ON.” To assess symptom experience, the SESS used a modified state version of the 34‐item Checklist for Symptoms in Daily Life (Wientjes and Grossman 1994; Witthöft and Rubin 2013). This modified version was used to provide consistency with our previous research (Verrender et al. 2018a; Verrender et al. 2018b; Verrender et al. 2025). Participants were asked “how strong/unpleasant are the following symptoms right now?” anchored with the terms “Barely Detectable” and “Maximum Severity.” The symptom responses (scored out of 100) of each of the 34 items were added to calculate a total symptom score for each of the baseline and exposure intervals in each trial separately. The primary dependent variable of symptom score in the provocation trial was calculated as the difference between the baseline and exposure total symptom scores (exposure interval minus preceding baseline interval); a difference score was used to minimize the influence of baseline variability.

#### 2.5.2 Other Questionnaires

A risk perception questionnaire (RPQ) comprising 4 questions was used to assess EMF risk perception from mobile‐phones and Wi‐Fi. The 40‐item State (Y1) Trait (Y2) Anxiety Index (STAI) (Spielberger et al. 1970), the 60‐item NEO Five‐Factor Personality Index (NEO‐FFI) (Costa and McCrae 1992), the 10 item Revised Life Orientation Test (LOT‐R) (Scheier et al. 1994), and the 14‐item Bortner Rating Scale (BRS) (Bortner 1969) were used to assess various aspects of personality. The RPQ, NEO‐FFI, LOT‐R, and BRS are beyond the scope of this paper and will not be discussed further.

### 2.6 Procedure

A participant information sheet was sent to people who responded to the study advertisements and contacted the researchers. This sheet informed participants that a proportion of the population report being sensitive to and/or experiencing a range of symptoms that they attribute to RF‐EMF, described some of the symptoms and devices people report being sensitive to, and explained that the scientific evidence has yet to establish a clear relationship between symptoms and exposure. The general aims of the study were also listed in the information sheet.

Once eligibility for the study had been determined via a telephone screening interview, participants were booked in for one testing session starting at 12 pm which lasted for approximately 3 h. Before arriving at the laboratory, participants were instructed to abstain from consuming caffeine, food, and drinks (including water) for 1 h, and alcohol for 8 h before the commencement of their session, as well as to abstain from making mobile phone voice calls for 2 h, and to obtain a full night's sleep (7–9 h) before the commencement of their session.

The timeline of the trial is outlined in Figure 1. Upon arrival at the laboratory, participants were greeted and taken to an air‐conditioned office where a verbal explanation of the study protocol was relayed to participants and informed written consent was provided. Participants then provided a first saliva sample (Time 1 Cortisol). To obtain a saliva sample, participants were instructed to allow saliva to pool in the mouth and then tilt the head forward gently, wrap their lips over the Saliva Collection Aid, and gently guide saliva through the Saliva Collection Aid into the cryotube. Once it was determined that participants understood the instructions for providing a saliva sample, the researchers left the room to allow the participants privacy, and all subsequent samples were taken without researchers being present. Once the saliva sample was collected, participants completed all of the personality questionnaires during an 18‐min period. After completion of the personality questionnaires, participants were taken to an air‐conditioned laboratory and were seated inside a Faraday cage. After 21 min from the first saliva sample collection had elapsed, a second saliva sample was taken (Time 2 Cortisol). Participants then watched the first 20 min of the control video. Following this, a third saliva sample was taken (Time 3 Cortisol) and participants completed a STAI‐Y1 and RPQ. Next, the first double‐blind provocation trial commenced. The provocation trial consisted of a 5‐min pre‐exposure baseline interval, followed by a 15‐min exposure interval (Sham or Active, depending on randomization and counterbalancing), followed by a 25‐min postexposure interval. During this time, participants completed the SESS 4 min into the Baseline interval, 14 min into the Exposure interval, and 24 min into the post‐exposure interval. A fourth saliva sample was then taken (Time 4 Cortisol), and participants completed the STAI‐Y1 and RPQ. Following this, participants watched the second 20 min of the control video and a fifth saliva samples was taken (Time 5 Cortisol). Participants then completed a second double‐blind provocation trial with the alternate exposure, where they completed the SESS 4 min into the Baseline interval, 14 min into the Exposure interval, and 24 min into the post‐exposure interval. A sixth saliva sample was then taken (Time 6 Cortisol), and participants then completed the STAI‐Y1 and RPQ again. At the conclusion of the second provocation trial, participants were guided out of the Faraday cage and asked if they had any concerns or questions about any aspects of the experiment. No participants reported any concerns about the experiment.

### 2.7 Statistical Analysis

#### 2.7.1 Data Normality and Effect Sizes

Data were analysed using IBM SPSS for Windows 29 (IBM, Armonk, New York). Data were inspected for normality via visual inspection and a series of Shapiro‐Wilk tests. These inspections revealed that the data was not normally distributed. To address this, the change in SESS symptom score data (SESS Exposure minus SESS Baseline) for each trial and the change in salivary cortisol response (T4 Cortisol minus T3 Cortisol; T6 Cortisol minus T5 Cortisol) was normalized using a Two‐Step approach (Templeton 2011). This approach involves first transforming each variable into a percentile rank, which creates a uniform distribution, and then applying an inverse‐normal transformation to the percentile ranks to form a normal distribution. A limitation of the two‐step approach to normalization is that it is only successful to the extent that the variable of interest is continuous in nature, and it is limited in its ability to transform continuous data that have a small number of levels, such as those measured on a 5‐ or 7‐point Likert scales (Templeton 2011). Therefore, where assumptions of normality were violated for the questionnaire data (which used Likert scales), non‐parametric tests were employed instead of transforming.

Test specific effects sizes are reported for each analysis, including Cohen's *d* for paired samples *t*‐tests, *r* for Wilcoxon Signed‐Ranks tests, and partial eta squared for 2 × 2 mixed analysis of variance (ANOVA).

#### 2.7.2 Preliminary Analysis

To test whether baseline salivary cortisol concentration differed between the two exposure conditions, a two‐tailed paired samples *t*‐test was used to compare the mean difference in baseline salivary cortisol concentration between the Sham and RF‐ON exposure conditions.

To test whether participants' belief of exposure differed between the two exposure conditions, a Wilcoxon Signed‐Ranks test was used to compare SESS belief score (Exposure minus Baseline) between the Sham and RF‐ON exposure conditions.

To test whether RF‐EMF exposure increased symptom score, a one‐tailed paired samples *t*‐test was used to compare SESS symptom score (Exposure minus Baseline) between the Sham and RF‐ON exposure conditions.

To assess whether state anxiety score (STAI‐Y1) may have confounded salivary cortisol concentration and to determine whether this score needed to be included as an additional independent variable in the hypothesis driven analysis, a series of Spearman's Rho correlations was used to test whether salivary cortisol concentration was related to state anxiety score at the time of sample collection.

The preliminary analyses have not been adjusted for Type 1 error, and therefore, any significant results must be treated cautiously.

#### 2.7.3 Hypothesis Driven Analysis

To determine whether being exposed to active RF‐EMF exposure increased salivary cortisol concentration, and whether any effect of RF‐EMF exposure on salivary cortisol response was related to sex differences, a 2 × 2 mixed ANOVA was used. The difference in salivary cortisol concentration was the dependant variable, and the main effect of RF‐EMF condition (Sham; RF‐ON), and the interaction of RF‐EMF exposure condition and sex (male; female) were assessed.

Given that only two, 1‐degrees of freedom tests were employed to test the hypotheses (effect of exposure; interaction of effect of exposure with sex), no adjustment for multiple comparisons is required.

## 3 Results

### 3.1 Preliminary Analyses

**Figure 2:**
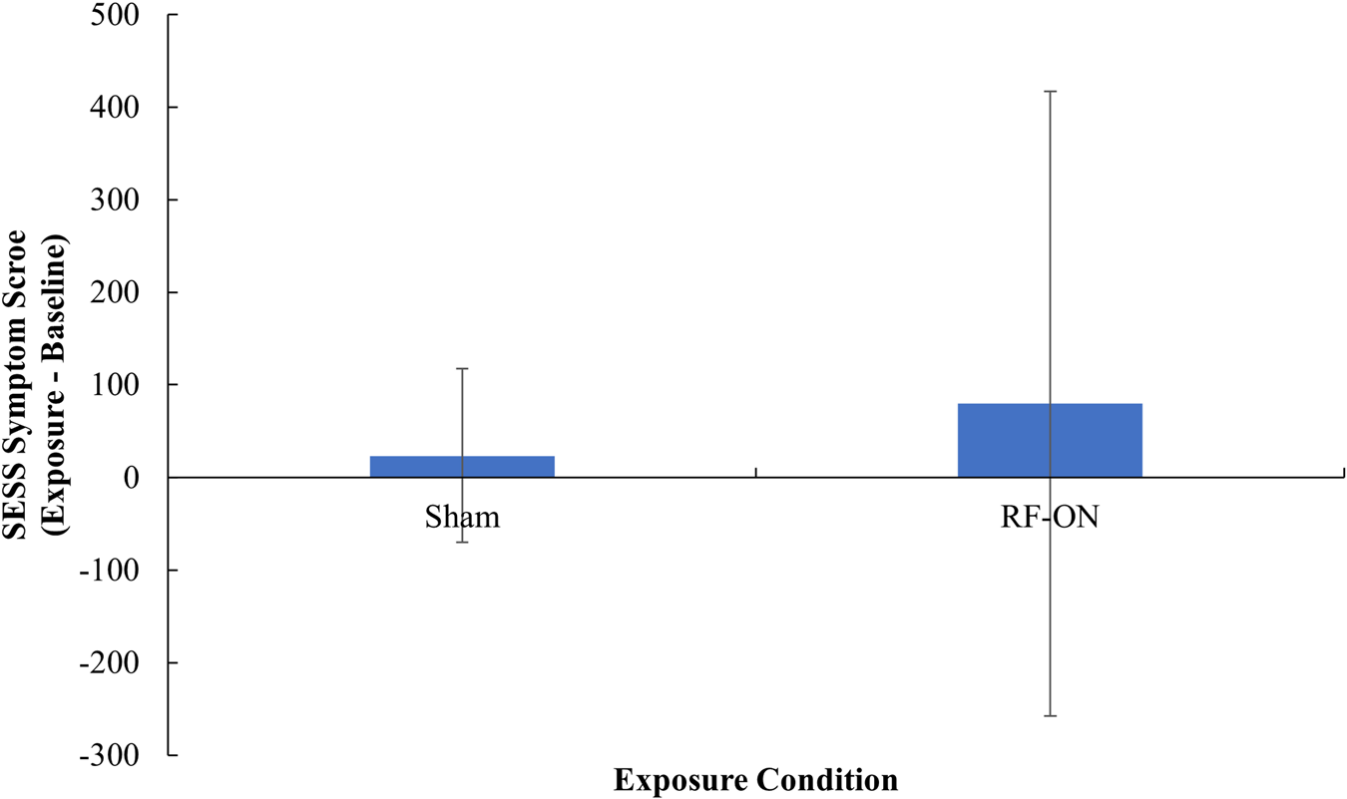
The mean change in SESS symptom score (Exposure − Baseline) as a function Exposure Condition. Data has been transformed using a two‐step normalization process. Error bars represent standard deviation.

No difference was found in salivary cortisol between the baselines before the Sham (*M* = 0.20, SD = 0.14) and RF‐ON (*M* = 0.19, SD = 0.09) exposure conditions, *t*(71) = −0.59, *p* = 0.554, *d* = −0.07. No difference was found in the change in SESS belief score between the Sham (*Mdn* = 26.50) and RF‐ON (*Mdn* = 46.00) exposure conditions, *Z* = −1.303, *p* = 0.192, *r* = −0.15. No difference was found in the change in SESS symptom score between the Sham (*M* = 23.61, SD = 93.49) and RF‐ON (*M* = 79.94, SD = 337.36) exposure conditions, *t*(71) = 1.46, *p* = 0.076, *d* = 0.26, as shown in Figure 2.

As shown in Table 1, the correlation coefficient between state anxiety (STAI‐Y1) score and salivary cortisol concentration was not statistically significant at any time point. Given this, state anxiety was not included as an additional independent variable in the subsequent statistical tests.

**Table 1:** Correlation between state anxiety score and salivary cortisol concentration.

Cortisol and STAI‐Y1 time point	Spearman's Rho	Significance
Cortisol T3 − State Anxiety T2	−0.019	0.875
Cortisol T4 − State Anxiety T3	−0.071	0.554
Cortisol T5 − State Anxiety T4	−0.100	0.402
Cortisol T6 − State Anxiety T5	−0.103	0.390

### 3.2 Hypothesis Driven Analysis

**Figure 3:**
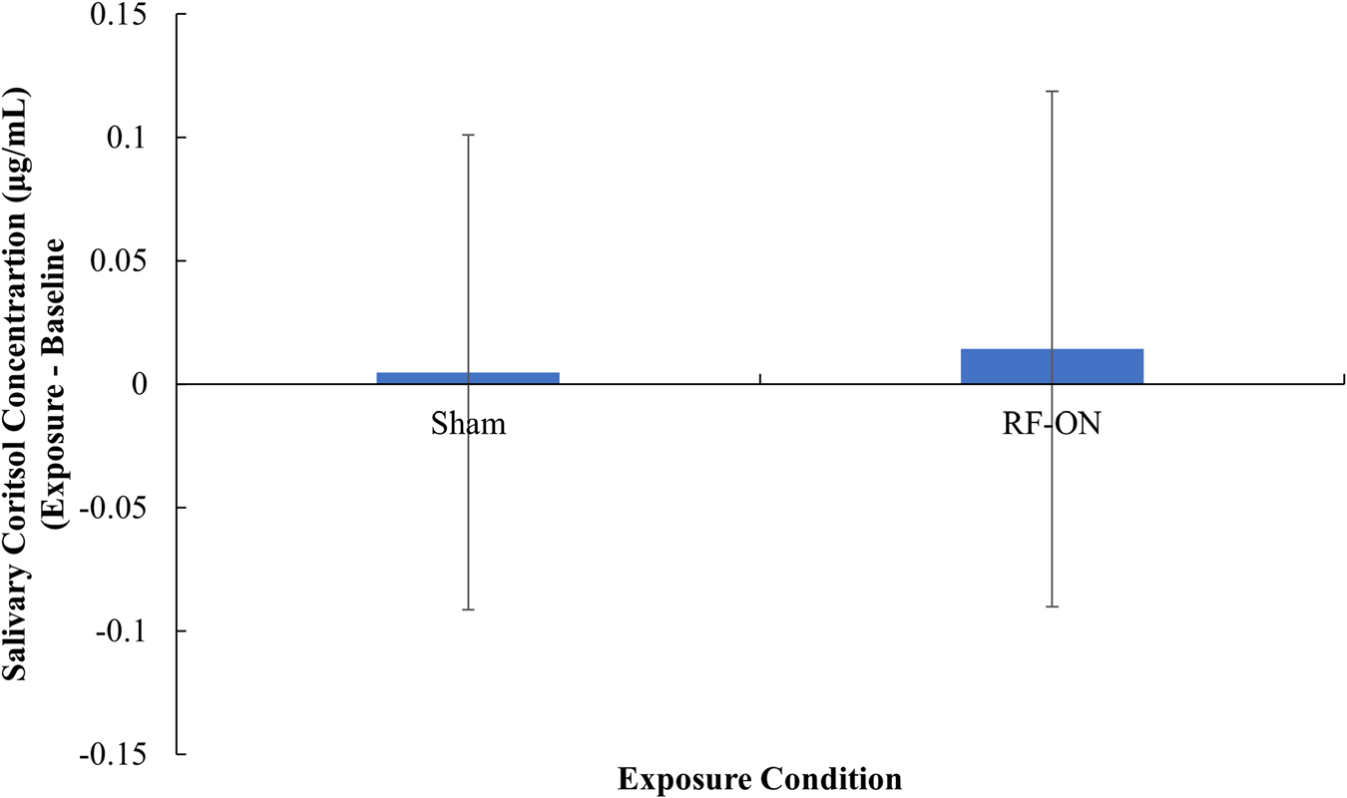
The mean change in salivary cortisol concentration (Exposure − Baseline) as a function Exposure Condition. Data has been transformed using a two‐step normalization process. Error bars represent standard deviation.

Table 2 shows the salivary cortisol concentration at each baseline and exposure interval by condition and sex. Figure 3 shows the change in salivary cortisol concentration as a function of exposure condition. No difference in the change in salivary cortisol between the Sham (*M* = 0.005, SD = 0.095) and RF‐ON (*M* = 0.015, SD = 0.105) exposure conditions was detected, *F*_(1, 70)_ = < 0.001 *p* = 0.987, ηp^2^ = <0.001. There was no interaction between participant sex and the change in salivary cortisol between the Sham and RF‐ON exposure conditions *F*_(1, 70)_ = 2.656, *p* = 0.108, ηp^2^ = 0.037.

**Table 2:** Salivary cortisol concentration (in μg/dL) in the Baseline and Exposure intervals for males (*n* = 24) and females (*n* = 48) in the Sham and RF‐ON exposure conditions. Data has been transformed using a two‐step normalization process and are presented as mean ± standard deviation.

	Sham		RF‐ON	
	Baseline	Exposure	Baseline	Exposure
Male (*n* = 24)	0.23 ± 0.13	0.03 ± 0.11	0.24 ± 0.09	<−0.01 ± 0.12
Female (*n* = 48)	0.19 ± 0.15	−0.01 ± 0.09	0.17 ± 0.08	0.02 ± 0.10

## 4 Discussion

The symptoms reported by individuals who experience IEI‐EMF can have substantial impact on social and occupational functioning and can considerably impair quality of life. While much of the existing evidence has demonstrated that these symptoms are the result of a nocebo effect, whether RF‐EMF exposure within the general public exposure limits influences underlying physiological processes that may result in symptoms remains unclear. Given the demonstrated relationship between low‐level RF‐EMF exposure and the engagement of thermoregulatory processes (Loughran et al. 2019), and the relationship between heat and free cortisol concentration in vitro (Cameron et al. 2010), there remains the possibility that cortisol concentration may be influenced by exposure to RF‐EMF. This potential physiological change may be one factor of a broad process of symptom development in IEI‐EMF, where multiple sources of both cognitive and physiological inputs first generate somatic sensations, which are then subsequently taken over and fostered by expectancy or learning‐based processes (Van den Bergh et al. 2021). However, while several studies have reported inconsistent, but mostly null effects of RF‐EMF exposure on cortisol, these studies suffer from several methodological issues. The current study was designed with several improvements to determine whether exposure to RF‐EMF increases salivary concentration, and to test whether any possible effect of RF‐EMF exposure on salivary cortisol was related to sex‐differences.

Preliminary tests first verified that there was no difference in baseline salivary cortisol concentration, participants belief of exposure, or change in symptom score between the Sham and RF‐ON exposure conditions, and that state anxiety score at the time of saliva sample collection did not confound salivary cortisol concentration. These preliminary tests were not only important for establishing that any subsequent differences in salivary cortisol between the two exposure conditions could be confidently attributed to the experimental manipulation and that state anxiety did not need to be included as an independent variable in the hypothesis‐driven analysis, but also to confirm that, consistent with the broader literature, active RF‐EMF exposure under double‐blind conditions did not increase symptoms in healthy participants.

The current study failed to find an effect of RF‐EMF exposure on salivary cortisol concentration, and there was no influence of participant's sex. While this is consistent with many previous studies (Andrianome et al. 2019; Braune et al. 2002; De Seze et al. 2001; Ghosn et al. 2015; Radon et al. 2001), one possible explanation for the lack of effect could be the assumed small‐to‐moderate effect size (*f* = 0.15) and limited power (0.8). However, this explanation seems unlikely given that the observed effect size for the main effect of RF‐EMF exposure on cortisol was negligible (effect size < 0.001), suggesting that the absence of a statistically significant effect was not due to insufficient statistical power. Moreover, given the novelty of the methodological improvements implemented in the current study, including controlling for diurnal variation in cortisol by only testing participants in the afternoon, attempting to capture a possible peak change in cortisol concentration by collecting samples between 21 and 40 min after the onset of a stimulus, using a 15‐min exposure protocol, and testing an adequate sample to detect small to moderate effects, we view this lack of effect as very robust.

There are, however, several possible limitations which may need to be considered in future research. First, the current study did not control for possible minor fluctuations in ambient temperature using a tightly controlled methodology such as a thermal perfusion suit. Given that the predications of the primary hypothesis were based upon the demonstrated effect of heat on CBG molecules in vitro, it is possible that fluctuations in ambient temperature may have confounded the results. However, the threshold at which ambient temperature begins to influence core body temperature is unlikely to have been reached in the current study. For instance, Henderson et al. (2021) found that core body temperature remains relatively stable during exposure to ambient temperatures up to 50°C (with 25% humidity), and this threshold is unlikely to have been met in the air‐conditioned laboratory used in the present study (circa 22°C). Nonetheless, in a previous study, we found that exposure to RF‐EMF increased finger temperature when ambient temperature was controlled using whole‐body thermal clamping (Loughran et al. 2019), suggesting that RF‐EMF exposure below the ICNIRP 2020 exposure limits induced a heating effect which resulted in vasodilation to the fingers. Although the stability of core‐body temperature during ambient temperature fluctuations reported by Henderson et al. (2021) suggests that it is unlikely that the lack of ambient temperature control (e.g., by using a thermal perfusion suit) affected the results of the current study, our previous study suggested that controlling for any possible thermal artifacts may be important for detecting small changes in physiology associated with RF‐EMF induced heating (Loughran et al. 2019). Second, the current study did not control for the possible effect of oral contraceptives. Given that women using oral contraceptives have been found to have increased CBG levels and lower salivary cortisol responses (Kumsta et al. 2007), it is possible that oral contraceptive use in the current sample may have decreased baseline salivary cortisol concentration and/or influenced the magnitude of any subsequent change in cortisol concentration due to RF‐EMF exposure. However, as data on oral contraceptive use was not collected in this study, it is difficult to determine the degree to which oral contraceptive use increased error variance. Finally, given that the current study tested predominantly young, healthy adults, the results cannot be generalized to other populations, including children, older adults, or those with poorer health. As the body's ability to thermoregulate is decreased by older age and chronic illness (Kenny et al. 2010), it is conceivable that the magnitude of any possible effect of RF‐EMF derived heating on CBG and cortisol concentration may be different across these populations. Furthermore, given that IEI‐EMF has been found to be more‐consistently reported by women over the age of 40 (Baliatsas et al. 2012), it may be important to test for any possible effect of RF‐EMF on cortisol concentration in this population. Similarly, it may also be useful to replicate the current results in a sample of IEI‐EMF individuals, in case they differ from a sample of young, healthy adults, in terms of their potential cortisol response to RF‐EMF.

Although the current study did not find any evidence of an RF‐EMF derived change in cortisol concentration in healthy participants, it provides a valuable direction for future research. Rather than testing for mere associations between any possible biomarker and subjective reports of ill‐health, it may be important to establish and test theoretically plausible interactions between low‐level RF‐EMF and the human body (i.e., known biophysical mechanisms). It remains possible that small, temperature‐induced changes in physiology caused by RF‐EMF exposure may be interpreted as one of several cognitive and physiological inputs that may lead to a somatic sensation. Given that low‐level RF‐EMF exposure has been found to trigger a thermoregulatory response (Loughran et al. 2019), it is conceivable that other physiological processes affected by heat may be influenced by RF‐EMF exposure. In this case, further research is required to determine whether the heat generated by RF‐EMF exposure is sufficient to influence other thermally mediated processes which could potentially trigger somatic sensations.

## Author Contributions

**Adam Verrender:** conceptualization, methodology, software, validation, formal analysis, investigation, data curation, resources, writing – original draft, writing – review and editing, visualization, supervision, project administration, funding acquisition. **Jacob Manley:** conceptualization, investigation, writing – review and editing. **Nikkeah K. Wallace:** investigation, data curation, writing – review and editing, project administration. **Sarah P. Loughran:** conceptualization, methodology, writing – review and editing. **Rodney J. Croft:** conceptualization, methodology, resources, writing – review and editing, supervision, funding acquisition.

## Conflicts of Interest

Sarah P. Loughran receives funding from The National Health and Medical Research Council of Australia (NHMRC). She is the Director of Radiation Research and Advice at the Australian Radiation Protection and Nuclear Safety Agency (ARPANSA), a member of the Scientific Expert Group at the International Commission on Non‐Ionizing Radiation Protection (ICNIRP), and a member of the World Health Organisation Task Group on Radiofrequency Fields and Health Risks. Rodney J. Croft receives funding from The National Health and Medical Research Council of Australia (NHMRC) and is a member of the Scientific Expert Group at the International Commission on Non‐Ionizing Radiation Protection (ICNIRP). No potential competing interests were reported by the remaining authors.

## Data Availability

The data that support the findings of this study are available from the corresponding author upon reasonable request.
